# A delivery system for field application of paratransgenic control

**DOI:** 10.1186/s12896-015-0175-3

**Published:** 2015-06-23

**Authors:** Arinder K. Arora, Adam Forshaw, Thomas A. Miller, Ravi Durvasula

**Affiliations:** Department of Biology, University of New Mexico, Albuquerque, NM 87131 USA; UNM School of Medicine, Albuquerque, NM 87131 USA; Department of Entomology, University of California, Riverside, CA 92521 USA; Department of Internal Medicine, Center for Global Health, UNM School of Medicine, Albuquerque, NM 87131 USA; New Mexico VA Healthcare System, Albuquerque, NM 87108 USA

**Keywords:** Paratransgenesis, Microencapsulation, Pierce’s Disease, *Xylella fastidiosa*, *Homalodisca vitripennis*, *Pantoea agglomerans*, Calcium-alginate

## Abstract

**Background:**

As an alternative to chemical pesticides, paratransgenesis relies on transformation of symbiotic bacteria of an arthropod vector to deliver molecules that disrupt pathogen transmission. For over a decade paratransgenesis has remained a laboratory-based endeavor owing to regulatory concerns regarding introduction of transformed microorganisms into the environment. To facilitate field application of paratransgenic strategies, risk mitigation approaches that address environmental contamination and gene spread must be developed.

**Results:**

Using biopolymer manipulation, we introduce a novel microencapsulation platform for containment and targeted delivery of engineered bacteria to the gut of a disease-transmitting arthropod. We demonstrate the first proof of principle of targeted delivery of EPA-approved *Pantoea agglomerans* E325 in a paratransgenic system to control spread of Pierce’s Disease by glassy-winged sharpshooters, (*Homalodisca vitripennis*) under simulated field conditions. Engineered microcapsules may address regulatory concerns regarding containment of recombinant bacteria and environmental spread of foreign genetic material and may represent an important step in translating paratransgenic science beyond the lab and into the field.

**Conclusions:**

We present, for the first time, a microencapsulation strategy to deliver recombinant bacteria to an insect and demonstrate targeted release of bacteria into the physiologically relevant region of the insect gut. This is a first step toward addressing concerns related to field application of recombinant bacteria. Engineered microparticles may decrease environmental contamination, horizontal gene transfer and competition with native species by acting as a barrier between recombinant bacteria and the environment.

**Electronic supplementary material:**

The online version of this article (doi:10.1186/s12896-015-0175-3) contains supplementary material, which is available to authorized users.

## Background

Arthropod-borne diseases remain a major threat to global health and exact a huge toll on agriculture and food security. These diseases are largely controlled through use of insecticides that reduce insect populations. Environmental applications of these chemicals may be associated with toxic residues and emergence of target insect resistance. Alternatives to insecticide-based control include paratransgenic manipulation of insects with genetically engineered bacteria that deliver transmission-blocking molecules to disrupt pathogens within the arthropod vector [1–7]. Though several paratransgenic insect systems are under development, field use of this strategy has not yet been realized, largely due to lack of delivery methods that target transformed bacteria to the arthropod while minimizing collateral spread of foreign DNA. The United States Environmental Protection Agency (EPA) has issued guidelines for introduction of genetically engineered (GE) species that state that GE organisms must: (a) Be contained to specific environments of introduction, (b) Not out-compete native species for resources, (c) Minimize foreign gene contamination (horizontal gene transfer) into native gene pools [8].

Here we report a novel strategy for delivery of genetically-engineered bacteria in a paratransgenic system that targets the glassy-winged sharpshooter (*Homalodisca vitripennis*), a pest of grapes and citrus that spreads the pathogen, *Xylella fastidiosa* [9–12]. Using simple and inexpensive materials for bioencapsulation [13–16] of the engineered symbiotic bacterium, *Pantoea agglomerans*, we demonstrate targeting of the sharpshooter, *H. vitripennis*, under controlled conditions with an alginate hydrogel that is tuned to release its bacterial payload during xylem flow into the foregut of the insect. By deploying a microencapsulation system that permits gated delivery of the bacterial payload to the arthropod, while greatly minimizing release in the environment, we believe that robust field-applicable technologies for paratransgenic control of arthropod-borne diseases will be possible.

## Results

### A paratransgenic model for the glassy-winged sharpshooter (GWSS)

**Fig. 1:**
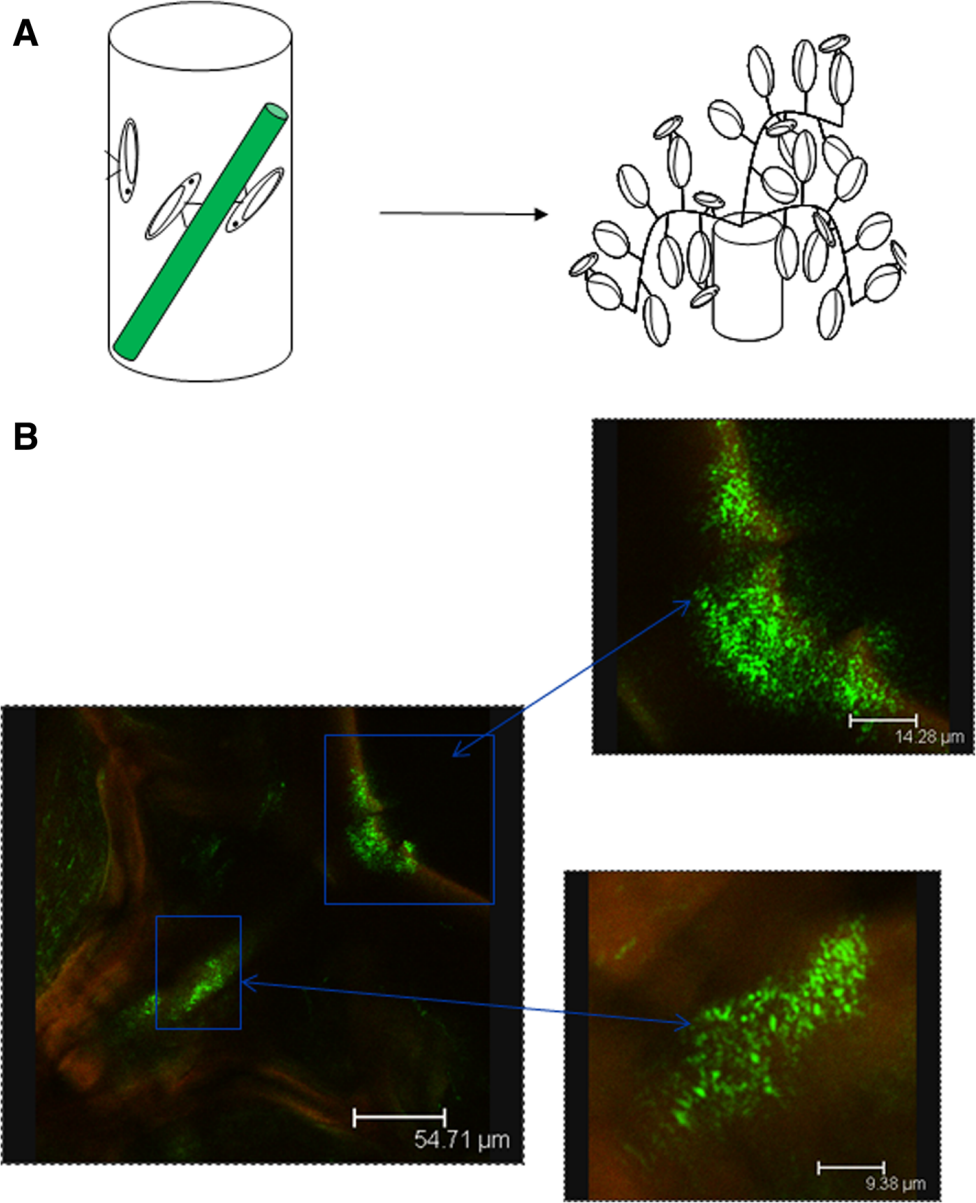
Glassy-winged sharpshooters acquired EGFP-expressing *P. agglomerans* from an artificial feeding system. (**a**) Glassy-winged sharpshooters were allowed to feed on *P. agglomerans* through artificial feeding system for 48 h. These insect were then kept on naive plants for 24 h to flush out the unbound bacteria from the foregut before dissection. (**b**) Image showing EGFP expressing *P. agglomerans* colonizing the foregut of GWSS. Inset: A higher magnification of same image showing *P. agglomerans* colonizing cibarium and precibarium of the GWSS

**Fig. 2:**
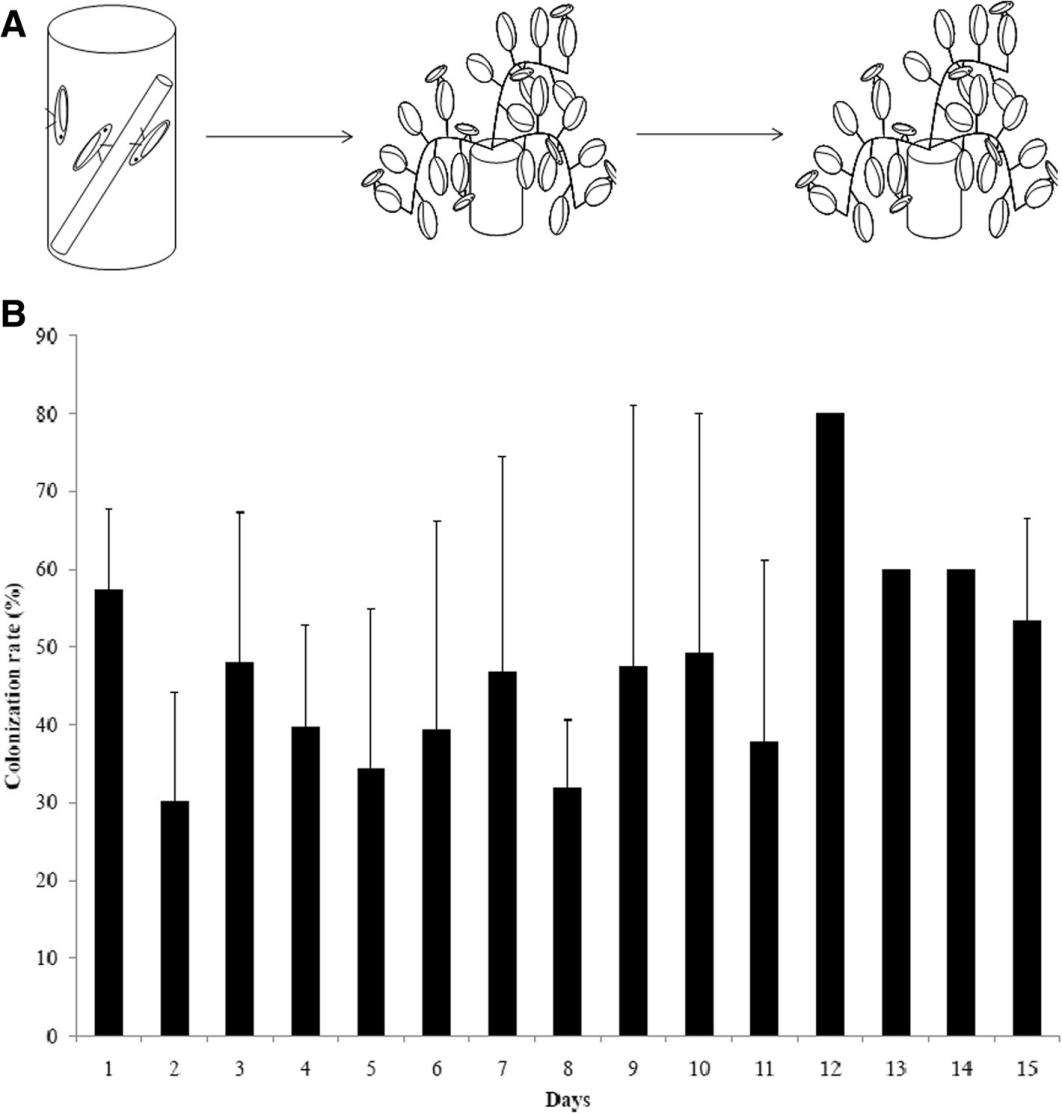
EGFP-expressing *P. agglomerans* persisted inside the foregut of the glassy-winged sharpshooter. (**a**) GWSS were fed EGFP-expressing *P. agglomerans* via an artificial feeding system for 48 h. The insects were moved to naive grape plant for 24 h to wash out unattached bacteria. They were subsequently housed on another naive grape plant for up to 15 days. Insects were collected daily, and processed as described. Homogenates were plated on LB plates to determine the presence of *P. agglomerans*. (**b**) Percent of GWSS carrying EGFP-expressing *P. agglomerans* over a period of 15 days. No difference in proportions of GWSS carrying EGFP-expressing *P. agglomerans* was observed. *p* = 0.1979 by chi-squared test for homogeneity

Pierce’s Disease (PD) of grapevines, caused by the bacterium *Xylella fastidiosa*, is a devastating disease of grapes that threatens grape production worth $3.2 billion and wine production worth $18.5 billion in California [17, 18]. Paratransgenic sharpshooters can be an answer to the threat posed by PD. Early attempts to establish paratransgenic sharpshooters were focused on the GWSS symbiont *Alcaligenes xylosoxidans denitrificans* [10]. However, concerns that this bacterium may be a nosocomial human pathogen led us to a grape endophyte bacterium, *Pantoea agglomerans* E325, currently approved by EPA for control of fire blight in apples and pears [19]. We hypothesized that the sharpshooter can acquire *P. agglomerans* in the same way as it acquires *X. fastidiosa*. To test this we genetically engineered this bacterium to express enhanced green fluorescent protein (EGFP) and ampicillin resistance and used an artificial feeding system (AFS) to deliver recombinant bacteria to GWSS [20]. The GWSS was able to acquire the engineered *P. agglomerans* from the artificial feeding system and EGFP-expressing *P. agglomerans* was found binding to the pre-cibarial and cibarial regions of the insect foregut (Fig. 1b). We further demonstrated that over a 15-day period, which is one third of the insect’s lifespan, EGFP-expressing *P. agglomerans* persisted in the insect foregut primarily colonizing the pre-cibarial and cibarial regions (Fig. 2b). We tested 522 sharpshooters over two seasons to study *P. agglomerans* persistence inside the GWSS foregut and observed 42.2 % foregut colonization with EGFP-expressing *P. agglomerans* over a period of 15 days. Carriage of *P. agglomerans* in the foregut of sharpshooters was not affected by time (*p* = 0.1979; chi-squared test). This confirmed that, once acquired by the insect, *P. agglomerans* persisted in the foregut for at least two weeks. Colonization with EGFP-expressing *P. agglomerans* occurs in the same spatial niche as the pathogen *X. fastidiosa* (Fig.1b) [21, 22], suggesting that *P. agglomerans* may be used as a delivery vehicle for paratransgenic control of Pierce’s Disease.

### Microencapsulation strategy for paratransgenic control

We developed a biopolymer microencapsulation system for paratransgenic manipulation of sharpshooters with 4 overall goals: (1) encapsulation of transgenic bacteria to keep them alive, contained and prevent horizontal gene transfer to environmental bacteria (2) release of bacterial payload from capsules tuned to specific physiologic conditions within the arthropod to minimize environmental contamination (3) release of bacteria from the capsules to colonize the target region of the sharpshooter where pathogens reside and (4) use of encapsulation materials that are inert in the insect without adverse physiological impact.

### A suitable polymer

**Fig 3:**
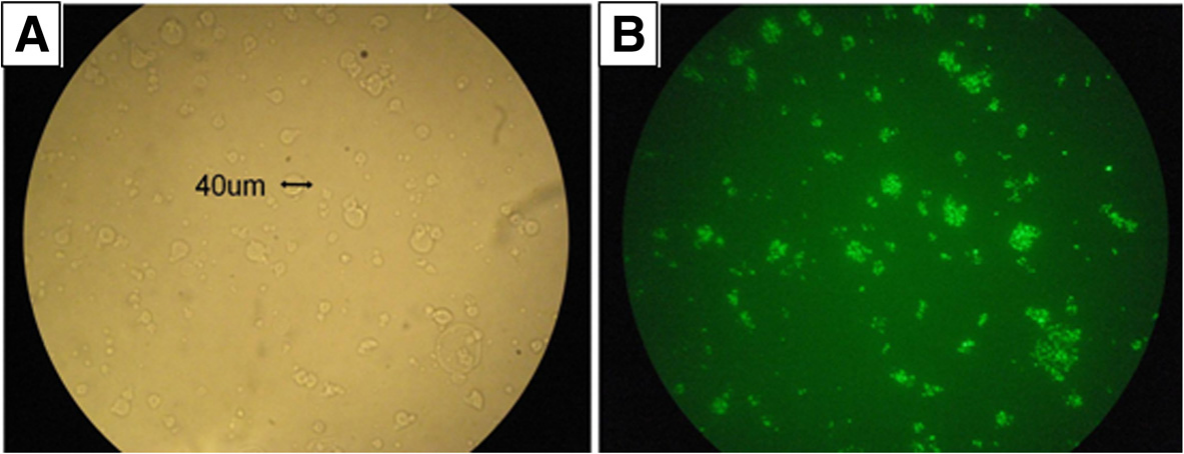
Microparticles containing EGFP-expressing *P. agglomerans*. (**a**) Calcium-alginate microparticles containing EGFP-expressing *P. agglomerans* (600×). (**b**) Same field under fluorescence. (600×)

We have developed a calcium-alginate microcapsule for containment of EGFP-expressing *P. agglomerans* (Fig. 3). To ensure that the particles would fit in the proboscis of the sharpshooter, we used a modified aerosolization-coascervation process to generate micron-sized particles. The particle size varied from 6–90 μm, with 57 % of particles in the range of 22–44 μm (Additional file 1: Figure S3). By altering the particle composition, we were able to achieve rapid and sustained release of bacterial payload in response to sudden hydration. Calcium-alginate based microparticles (in absence or presence of glycerol) resulted in sustained release of bacteria over a period of 7 days in response to hydration, which was significantly higher than calcium/barium-alginate microparticles (Additional file 2: Figure S4, *p* < 0.0001). Maximum release of *P. agglomerans* (CFUs = 1.03X10^5^) was observed from calcium-alginate microparticles with 10 % glycerol over a period of 7 days, while minimum (CFUs = 7.33) release was observed from calcium/barium-alginate microparticles. Based on these results we decided to engineer calcium-alginate based microparticles for acquisition experiments to achieve a quick as well as sustained release of bacteria inside the insect foregut after acquisition of microparticles by the sharpshooters during xylem feeding.

**Fig. 4:**
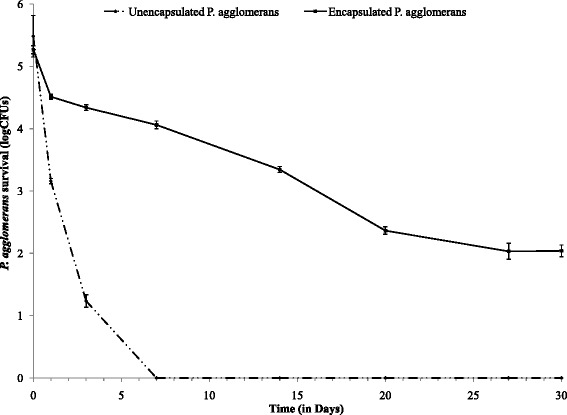
Bacterial survival under extreme dehydration. Encapsulation of *P. agglomerans* significantly increases bacterial viability compared to unencapsulated controls during desiccating conditions. Unencapsulated *P. agglomerans* died in a week after dehydration, while encapsulated *P. agglomerans* survived for a month

The encapsulation process protected *P. agglomerans* from desiccating conditions, which are expected during periods of coating on plants prior to ingestion by the sharpshooter (Fig. 4). Under extreme desiccation 100 % of unencapsulated *P. agglomerans* died within 7 days; on the other hand only 2 log decrease in colony forming units (CFUs) was observed when encapsulated *P. agglomerans* were exposed to desiccation for the same time period (Fig. 4, *p* < 0.001). We further observed that calcium-alginate microparticles were able to keep *P. agglomerans* alive for 30 days, though the number of CFUs in microparticles decreased over time. This survival of bacteria during desiccation is important, as similar conditions are expected during summer in grape growing areas of Southern California.

**Fig. 5:**
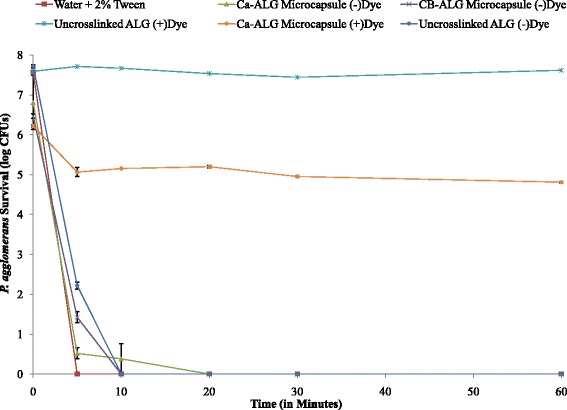
*P. agglomerans* survival after exposure to UVC light. Encapsulated and unencapsulated *P. agglomerans* in the absence of dye were killed after exposure to UVC within 20 min., while both encapsulated and unencapsulated *P. agglomerans* in the presence of dye were able to withstand UVC exposure for 60 min

Addition of 0.5-1.0 % of India ink to the particle confers high-level UV-resistance, again a desirable quality during periods of application on plants in the summer time (Fig. 5). Exposure of both encapsulated and unencapsulated *P. agglomerans* in the absence of India ink to UVC resulted in 100 % killing in 20 min. There was no significant reduction in CFUs over a period of 60 min when *P. agglomerans* mixed with alginate and India ink were exposed to UVC (Fig. 5, *p* = 0.051). However, when Ca-alginate microparticles containing India ink were exposed to UVC there was one log reduction in *P. agglomerans* CFUs in the first 5 min, (Fig. 5) and CFU numbers remained steady thereafter for next 55 min. We speculate that bacteria present on the surface of microparticles were exposed to UVC and were killed in the first 5 min of exposure, but the bacteria embedded in the microparticles remained protected.

We believe that calcium-alginate-10 % glycerol hydrogel particles with India ink, which protected against dehydration and UVC, are ideally suited to deliver transgenic *P. agglomerans* under field conditions to the glassy-winged sharpshooter. We hypothesized that these particles will swell during xylem flow and release the bacterial payload into the foregut of feeding insects.

### A proof-of-concept

**Fig. 6:**
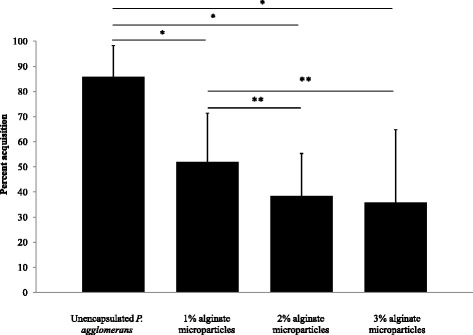
Glassy-winged sharpshooters acquired EGFP-expressing *P. agglomerans* from calcium alginate microparticles. Percent glassy-winged sharpshooters carrying EGFP-expressing *P. agglomerans* in their foregut. A higher percent of GWSS acquired *P. agglomerans* from plants painted with unencapsulated *P. agglomerans* than from plants painted with encapsulated *P. agglomerans*.* *p* < 0.05 by chi-squared test for homogeneity.** *p* > 0.05 by chi-squared test for homogeneity

**Fig. 7:**
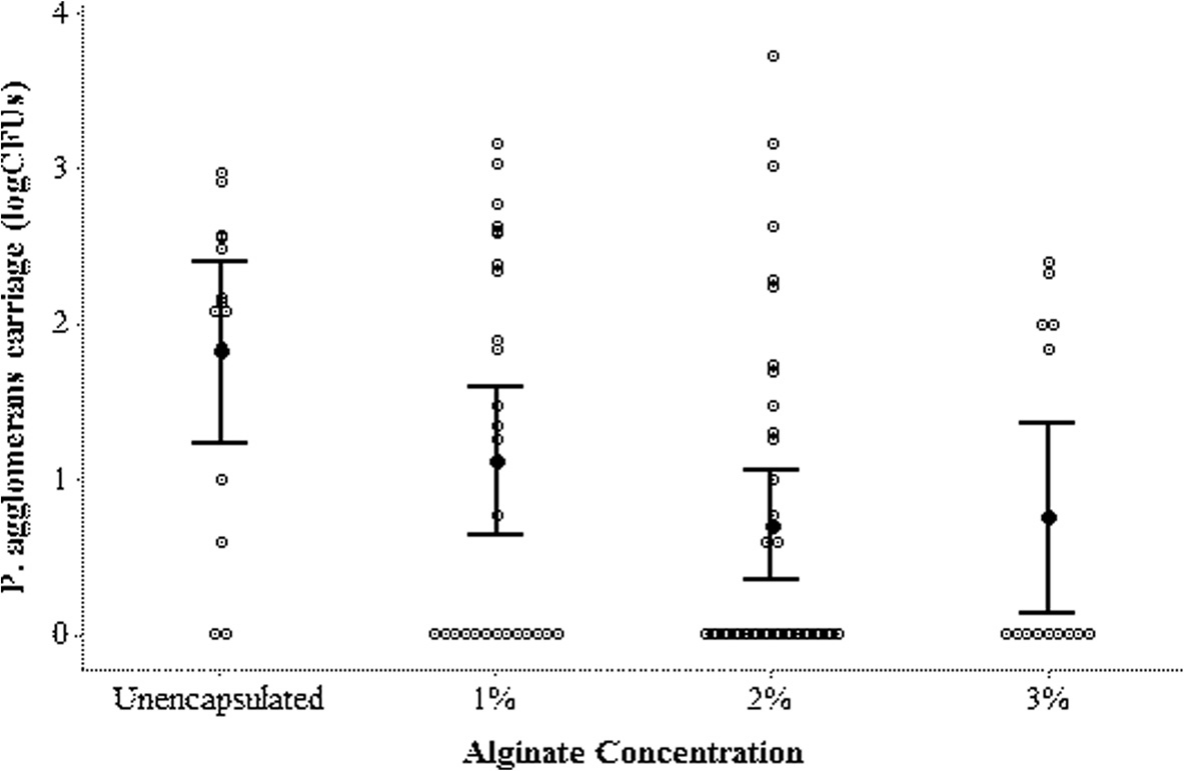
Bacterial CFUs per insect head. *P. agglomerans* (EGFP-expressing) colony forming units (log10) per insect head. No difference in *P. agglomerans* CFUs acquired by the GWSS from plants painted with unencapsulated or encapsulated *P. agglomerans* was observed. *p* > 0.05 by one-way ANOVA with Tukey Simultaneous Tests for means with unequal variance

Glassy-winged sharpshooters were able to acquire *P. agglomerans* from alginate-based microparticles painted on grape plants. In a control group, GWSS fed on plants painted with unencapsulated *P. agglomerans*, 85.7 % of the insects acquired *P. agglomerans*, which was significantly higher than the number of GWSS that acquired *P. agglomerans* from plants painted with encapsulated *P. agglomerans* (Fig. 6, *p* = 0.016). Amongst the groups of sharpshooters fed on encapsulated *P. agglomerans* maximum colonization was observed in insects acquiring *P. agglomerans* from 1 % alginate-based microparticles (51.8 %) followed by 2 % (38.5 %) and 3 % (35.7 %) alginate-based microparticles. Though an increase in alginate concentration decreased *P. agglomerans* acquisition by the GWSS, it was not statistically different between three tested concentrations of alginate - 1, 2 and 3 % (Fig. 6, *p* = 0.476). Unencapsulated treatments resulted in the maximum CFUs per insect head (mean CFUs = 245.4 ± 80.4) and minimum CFUs per head were observed in treatments using 3 % alginate microcapsules (mean CFUs = 52.1 ± 22.7) (Fig. 7). We did not observe significant difference between Pantoea CFUs acquired by the insects from microparticles engineered with 1, 2 or 3 % alginate (Fig. 7, *p* = 0.33).

Though the association between alginate concentration and Pantoea acquisition did not attain statistical significance, there was a trend toward decreased Pantoea levels in GWSS with increasing alginate in microparticles. This suggests that manipulation of polymer composition may alter levels of bacterial colonization in the sharpshooter foregut.

## Discussion

Here, we report a new paratransgenic strategy for control of Xylella transmission by the glassy-winged sharpshooter, *H. vitripennis. P. agglomerans* E325, an EPA approved agent for biocontrol of certain crop diseases, has been genetically modified using plasmid pT3078, which has the *bla* gene as a marker, to express recombinant EGFP in the foregut of GWSS. We have demonstrated robust EGFP expression in the insect foregut, in the region that harbors the plant pathogen, *X. fastidiosa*. Furthermore, we report the use of a simple and inexpensive microencapsulation strategy to contain engineered Pantoea and release the modified bacteria during conditions of xylem flux into the anterior mouthparts of the feeding insect. Our results represent the first iteration of this paratransgenic delivery system. Refinement of polymer chemistry and capsule application may increase the percentage of GWSS that acquire transgenic Pantoea in future trials. Chemical functionalization of the polymer backbone to enhance bacterial release along with refined methods of increasing bacterial CFUs per capsule are currently underway.

We propose the use of an EPA approved biocontrol agent, *P. agglomerans* E325*,* as a paratransgenic control vehicle for PD. *P. agglomerans* colonized the foregut of the insect from both an artificial feeding system and plants painted with unencapsulated and encapsulated bacteria. *P. agglomerans* persisted in the insect foregut over the entire duration of testing, up to15 days. The 15 -day period is significant since it represents close to one third of the insect’s lifespan. Additionally, field applications of this approach could involve bi-weekly delivery of encapsulated bacteria to grape plants, thus offering fresh inocula to GWSS on a regular basis. *P. agglomerans* can be engineered to produce anti-Xylella molecules such as antimicrobial peptides (AMPs) or anti-Xylella antibodies to block *X. fastidiosa*. We have used plasmid pT3078, which has the *bla* gene as a marker in addition to an EGFP gene. However, for field application, Pantoea lines expressing anti-Xylella molecules without antibiotic marker genes will be engineered. *P. agglomerans* lines with fluorophore genes or REDantibody genes as markers are under development for field release. These strategies, coupled with a refined delivery system, will produce a robust toolset in the battle against GWSS-mediated PD transmission.

Field use of paratransgenic control for arthropod-borne diseases remains a future prospect, largely due to concerns about the fate of engineered bacteria. In the current model, we believe that environmental risks associated with release of foreign genes will be reduced. First, plasmid decay for the recombinant *P. agglomerans* (Additional file 3: Figure S2) occurs at the rate of 0.0533 % per bacterial generation; thus engineered bacteria are expected to revert to wild-type forms. Furthermore, engineered *P. agglomerans* grows at a rate comparable to wild-type counterparts (Additional file 4: Figure S1), suggesting that it cannot out-compete the native organism. Second, we have encased the recombinant symbiont in an alginate particle that inhibits release of payload into the environment. Physical contact between engineered bacteria and native bacteria of the rhizosphere or phytosphere is greatly impeded by the presence of capsules. Thus horizontal gene transfer between engineered *P. agglomerans* and commonly present bacteria of environmental consortia - a very rare event that occurs in the 10^−8^ to 10^−9^ range (data not shown) - should be further diminished. The overall aim of this strategy is to reduce horizontal gene transfer to levels that are acceptable to regulatory agencies, rather than eliminating gene flow. Abolishing gene flow between environmental bacteria is an unrealistic goal. However, by providing a physical barrier via microparticles we can minimize exchange between the large number of freshly released bacteria used in plant inundation and environmental microbes, with the aim of reducing unwanted gene flow.

We have contained engineered *P. agglomerans* using calcium-alginate microparticles. To deliver the bacteria to GWSS, we have chosen to paint growing grape shoots, thus facilitating ingestion of the organisms during insect feeding. GWSS and other sharpshooters are xylem feeders [21]. They initially contact a plant surface with the tip of their stylet before penetrating into xylem vessels [22]. We hypothesize that bacteria-laden microparticles that have been painted on plant shoots will contaminate the lumen of the insect’s stylet. During the sucking of xylem, hydrostatic pressure gradients as high as 0.24 MPa/cm [23], will cause rapid swelling of the alginate hydrogel and release Pantoea into the cibarial region of the sharpshooter’s foregut. To optimize release of bacteria from the microcapsules and enhance colonization of the insect, hydrogels may be tuned by varying the composition and concentration of divalent cations (Ca^++^, Ba^++^) as well as alginate (Figs. 6 and 7).

Over 80 % of GWSS that were exposed to unencapsulated (naked) Pantoea during feeding became colonized with the bacteria, at a mean CFU value of 245.4 ± 80.4. (Figs. 6 and 7). We tested microparticles containing 1 %, 2 % and 3 % alginate (w/v) in the hydrogel. In all cases, there was a significant reduction in Pantoea acquisition when compared to the control group (GWSS exposed to unencapsulated bacteria) (Fig. 6). When an alginate concentration of 1 % (w/v) was used, target GWSS acquired Pantoea at a rate of 51.8 % with a mean CFU count of 186.6 ± 68.9. This rate of acquisition was the highest amongst the tested alginate concentrations and we believe that 1 % alginate particles merit further attention. Rates of acquisition of encapsulated Pantoea by target GWSS may have been lowered for several reasons. First, it is likely that the experimental insects represented a heterogeneous population. It is difficult to maintain homogeneous GWSS population under controlled conditions, thus, we used field-collected insects that were previously colonized with environmental bacteria, genetically distinct and of varying ages through the course of the summer. Second, we used a very limited time frame for acquisition of bacteria through feeding by GWSS. It is likely that future field applications that employ multiple treatments of plants with greatly protracted contact time between GWSS and particles will increase colonization rates in the insect. Third, many refinements in particle composition are possible. We are exploring use of particles made of other materials that would dissolve in the presence of enzymes present in the insect saliva to better release the bacterial payload. Attractants and feeding stimulants may be incorporated to enhance bacterial delivery. Indeed, future versions of the microencapsulation-delivery system should result in higher rates of target insect colonization with engineered Pantoea.

Microencapsulation is a first step toward addressing concerns associated with paratransgenic manipulation of arthropods. In this proof of principle, we deployed microcapsules to deliver a recombinant bacterium to an insect. We designed the alginate particles to swell under very high hydrostatic pressures encountered during xylem flux and to decrease contact between engineered bacteria and the environment during periods of application and residence on plants. Microencapsulation will decrease direct interactions of released bacteria with other epiphytes when compared to unencapsulated bacteria. This should also decrease the interactions of released bacteria with rhizosphere organisms. We believe this is a first step toward targeted delivery of recombinant bacteria to arthropod vectors of disease. In ongoing projects, we are designing more advanced particles that respond to specific physiological cues within insects, such as pH and blood products, to establish a platform for field use of paratransgenics.

Based on this technology, microcapsule-based delivery of transgenic bacteria to arthropods of clinical significance (sand flies, kissing bugs, mosquitoes) for human vector-borne disease prevention becomes a real and logical extension, signifying a paradigm shift in paratransgenic techniques from the lab to field settings. A microparticle-based recombinant-bacterial release strategy could make paratransgenic control of Chagas disease and malaria - for which paratrangenic manipulation has been shown to decrease parasite load inside the vector - and other vector-borne diseases a reality.

## Conclusion

This is the first example of the use of microencapsulation to deliver recombinant bacteria to an insect gut. We have demonstrated that transgenic symbiotic bacteria can be delivered to the appropriate physiological niche within a disease-transmitting arthropod. Furthermore, by acting as a barrier between transgenic bacteria and the outer environment these microparticles should reduce competition between the recombinant bacteria and native species, environmental contamination and horizontal gene transfer. This platform may be expanded to deliver recombinant bacteria to other disease-transmitting arthropod vectors, thus facilitating field use of paratransgenic control.

## Methods

### Bacterial culture

*P. agglomerans* E325 culture was maintained on Luria Bertani (LB) agar at 30 °C or in LB broth at 30 °C at 180 rpm.

### Preparation *P. agglomerans* E325 competent cells

Bacterial cells were grown in 100 ml of LB medium at 30 °C with shaking at 175–180 rpm. When OD600 was reached 0.4-0.6 the cells were harvested by centrifugation at 4000 rpm for 10 min at 4 °C. These cells were resuspended in 40 ml of ice cold Inoue solution and harvested by centrifugation at 4000 rpm for 10 min at 4 °C. Bacterial cells were then resuspended in 10 ml of Inoue transformation buffer. To this solution 0.7 ml of DMSO was added and the cells were stored in ice for 10 min. Aliquots of 70 μl were made in microcentrifuge tubes and immediately snap frozen in liquid nitrogen. The competent cells were then stored at −80 °C.

### Transformation of *P. agglomerans* E325 with pT3078-5

Competent *P. agglomerans* cells were removed from −80 °C freezer, thawed and kept on ice for 10 min. Two μl of plasmid pT3078-5 was added to the cells. pT3078-5 has an EGFP gene and bla gene (ampicillin resistant) as markers. The cells were incubated on ice for 30 min followed by a heat shock at 42 °C for 90 s. The tubes were then transferred to ice for 2 min. The cells were recovered by adding 800 μl LB medium and incubated at 30 °C with shaking at 180 rpm for 2 h. These cells were then transferred on to the LB agar supplemented with carbenicillin (100 mg/liter of media) and incubated overnight at 30 °C. Next day single colonies were plated on LB agar with carbenicillin. Transformed *P. agglomerans* cells were tested for florescence and confirmed by EGFP gene PCR.

### Growth comparison

Wild-type *P. agglomerans* and EGFP-expressing *P. agglomerans* were grown overnight in LB and LB + carbenicillin, respectively, to a stationary phase. They were then diluted to 1/100 in 50 ml LB and were incubated at 30 °C in a shaker incubator at 200 rpm. OD600 readings were taken hourly until cultures reached stationary phase.

#### *Invitro* plasmid stability

Transformed *P. agglomerans* were grown in LB supplemented with carbenicillin for 10 h at 28 °C with shaking at 175–180 rpm. These cells were diluted to ~1000 cells/ml and were grown for 10 h (approx. 15 generation) in 25 ml LB broth without antibiotic selection. After 10 h of growth log phase cells were added to 25 ml fresh LB broth to a final concentration of ~1000 cells/ml and were grown again. This procedure was repeated 5 times (total of 75 generation). Every 10 h, while transferring the cells to fresh medium, a fraction of cells was aliquoted and grown overnight on LB agar without any selection. The next day 100 random colonies were selected and streaked on LB agar supplemented with carbenicillin.

### Acquisition of *P. agglomerans* to the GWSS using artificial feeding system

The EGFP plasmid containing *P. agglomerans* was grown overnight in LB broth supplemented with carbenicillin. The next morning bacteria were centrifuged at 4000 rpm for 10 min followed by washing twice with the PBS. The bacterial cells were then resuspended in a solution of PBS (0.2×) and sugar (0.2 %). This solution was filled into plastic tubes with inner and outer diameter 1/8″ and 3/16″ , respectively. These tubes were placed in 2.5″diameter vials individually. The vials containing the tubes were called the artificial feeding system (AFS). The control AFS tubes were filled with the PBS-sugar solution without bacteria. GWSS, collected from the citrus orchard of Agriculture Operations, UC, Riverside, were put into the control AFS and the bacteria containing AFSs. These insects were given an acquisition access for 48 h.

### Maintenance of sharpshooters

After an acquisition access of 48 h the insects were transferred to an insect cage containing grape plants for 24 h to flush out the unattached bacteria. After 24 h of flushing, the insects were shifted to a new insect cage with clean grape plants. The control (naïve) insects from the acquisition experiment were also transferred to the clean plants. Everyday 5–15 insects were taken out from the cage for the bacterial isolation.

### Isolation of *P. agglomerans* from insect heads

Everyday 5–15 sharpshooters were taken out from bacteria and control groups and surface sterilized in 70 % ethanol and 10 % bleach followed by washing twice with autoclaved sterilized water. The heads of surface sterilized insects were cut with a sterile surgical blade and were homogenized in 100 μl PBS using a homogenizer. This solution was plated on carbenicillin supplemented LB agar plates.

### General materials and method for alginate experiments

Protonal LF10-60 alginate (FMC Biopolymer, Sweden) was prepared at 1-3 % (w/v) concentration in de-ionized water and autoclaved prior to use. Bacterial cultures were grown in LB broth overnight at 30 °C to plateau phase in 15 ml culture tubes, pelleted at 4000 RPM at 4 °C for 15 min, washed twice with sterile 1× PBS, re-pelleted at 4000 RPM and supernatant decanted and re-suspended in 3 ml sterile 1× PBS before incorporating into the alginate. Bacterial-alginate suspensions were utilized immediately after mixing. All experiments were performed in triplicate. Viable colony forming units (CFUs) were evaluated using the pour plate method on selection agar. Particle sizing was accomplished on a Microtrac s3500 laser diffraction analyzer (Microtrac Systems).

### Synthesis of calcium-alginate (Ca-ALG) or calcium/barium-alginate (CB-ALG) microparticle

Alginate-bacterial suspension was produced as mentioned above. The resulting mixture was atomized from an alcohol-sterilized airbrush into a vat containing sterile 0.05 M CaCl_2_ with constant agitation from a distance of 20 cm. The resulting microparticles were allowed to harden for 45 min and then harvested via vacuum filtration through a sterile Nalgene filter-funnel with two pieces of sterile Nylon mesh (pore sizes of 50 μm and 35 μm respectively) acting as filters. The microparticles were harvested from the mesh and stored for future use.

In the case of CB-ALG microparticles, an equal volume of BaCl_2_ was added to the CaCl_2_ solution after the 45 min gel time and the microparticles were allowed to further gel for an additional 20 min. Microparticles were then collected as described.

### Synthesis of carbon-calcium-alginate (CC-ALG) microparticles

For all experiments relating to carbon-calcium alginate particles, the alginate-bacterial suspension was mixed with 1 % vol/vol of sterile India Ink prior to particle gellation. The rest of the protocol was left unaltered.

### Dehydration of Ca-ALG microparticles

Bacteria-containing Ca-ALG were produced as previously described. Ten gram *P. agglomerans* Ca-ALG microparticles (1X10^7^ CFUs/g) or 10 gm *P. agglomerans* in 5 % glycerol (*v/v*) slurry (1X10^7^ CFUs/g) were then under sterile conditions transferred into sterile 10 cm petri dishes and allowed to dry to constant weight under ambient conditions and atmosphere (27 °C, 5-7 % RH) overnight. Over the course of several weeks at various time points 1 gm of dry slurry or dehydrated microparticles was transferred into fresh sterile 0.15 M sodium citrate and allowed to rehydrate/dissolve for 1 h and then serially diluted and plated on selection agar and incubated overnight. Viable CFUs were assessed based on colony count.

### Survival of encapsulated *P. agglomerans* E325 under highly sterilizing UVC conditions

Bacteria-containing Ca-alginate microparticles both with and without India Ink dye were produced as previously described. Ten grams of encapsulated *P. agglomerans* E325 containing 2.0 % (*v/v*) India Ink were placed in sterile open petri dishes 30 cm directly under a columnated UVC germicidal lamp. Samples were exposed to sterilizing radiation for 0–60 min, during which samples were chosen at random at various time intervals and placed in fresh sterile microcentrifuge tubes containing 1 ml sterile 0.15 M sodium citrate solution and left to dissolve over 1 h. The liberated microbes were serially diluted and plated on LB agar and left to incubate at 30 °C overnight. CFUs were counted the following day.

### Diffusion from CA-ALG and CB-ALG microparticles

Bacteria-laden microparticles were produced as previously described. 1 g of microparticle slurry was mixed with 1 ml of sterile water in a 1.5 ml microcentrifuge tube and allowed to stand at 4 °C for the prescribed time. At various time intervals throughout the experiment, the tubes were vortexed vigorously for 10-15 s to re-suspend any diffused bacteria. The microparticles were allowed to settle for 5 min and then 50 μl aliquots were removed and serially diluted. 50 μl aliquots of each dilution were then plated on selection agar and the plates were incubated overnight at 30 °C to determine viable CFUs that had diffused from the microparticles.

#### *P. agglomerans* acquisition through microparticles

EGFP-expressing *P. agglomerans* containing microparticles were engineered using a 1, 2 or 3 % alginate as described above. Encapsulated bacteria were irradiated three times with UVC for 90 secs to kill any unencapsulated bacteria during the production process. These microparticles were then mixed with 10 % glycerol and 3 % guar gum and were applied to the grape plants. Field collected sharpshooters were allowed to feed on these plants for 24 h. As a control unencapsulated bacteria mixed with 10 % glycerol and 3 % guar gum were applied to the grape plants. After 24 h the sharpshooters were removed from the plants and surface sterilized. The foregut and midgut contents were homogenized as described above using a homogenizer. The foregut and midgut contents were plated on LB + carbenicillin agar plates and were incubated at 30 °C. The CFUs were counted after 24 h.

### Statistical analysis

Number of GWSS carrying *P. agglomerans* were compared using Chi-squared test for homogeneity. *P. agglomerans* CFUs in various experiments with different treatments were analyzed by Tukey’s test for multiple comparison after taking log values of CFUs. All the values are shown as mean ± S.E. Statistical analysis were performed using Minitab version 17 for windows 7. *p* values < 0.05 were considered significant.

## Additional files

Additional file 1: Figure S3. Size distribution of *P. agglomerans* containing microparticles. Additional file 2: Figure S4. Diffusion of microencapsulated *P. agglomerans* from Ca^2+^ and Ca^2+^/Ba^2+^ cross-linked microparticles. Use of barium as a cross-linker greatly reduced the diffusion of bacteria from the microparticles.*, **, *** and **** *p* < 0.001 by one-way ANOVA with Tukey Simultaneous Tests for means with unequal variance compared to Ca-Alg and Ca-Alg + 10 % Glycerol microparticles on days 1, 3, 5 and 7, respectively. Additional file 3: Figure S2. Loss of plasmid pT3078-5 by *P. agglomerans* over time. Additional file 4: Figure S1. Comparison of growth of wild type *P. agglomerans* and EGFP-expressing *P. agglomerans*.

## Abbreviations

GWSS: Glassy-winged sharpshooter
EGFP: Enhanced green fluorescent protein
AFS: Artificial feeding system
EPA: Environmental Protection Agency
GE: Genetically engineered
PD: Pierce’s Disease
CFU: Colony forming unit
UVC: Ultraviolet C
AMP: Antimicrobial peptides
LB: Luria Bertani
Ca-ALG: Calcium alginate
CB-ALG: Calcium/barium alginate
CC-ALG: Carbon calcium alginate
