# Supplementary material for: A delivery system for field application of paratransgenic control
Source: BMC Biotechnol. 2015 Jun 23;15:59. doi: 10.1186/s12896-015-0175-3 (PMC4477610; doi:10.1186/s12896-015-0175-3)
Supplement: Additional file 1: Figure S3. — Size distribution of P. agglomerans containing microparticles. [file 12896_2015_175_MOESM1_ESM.pdf]

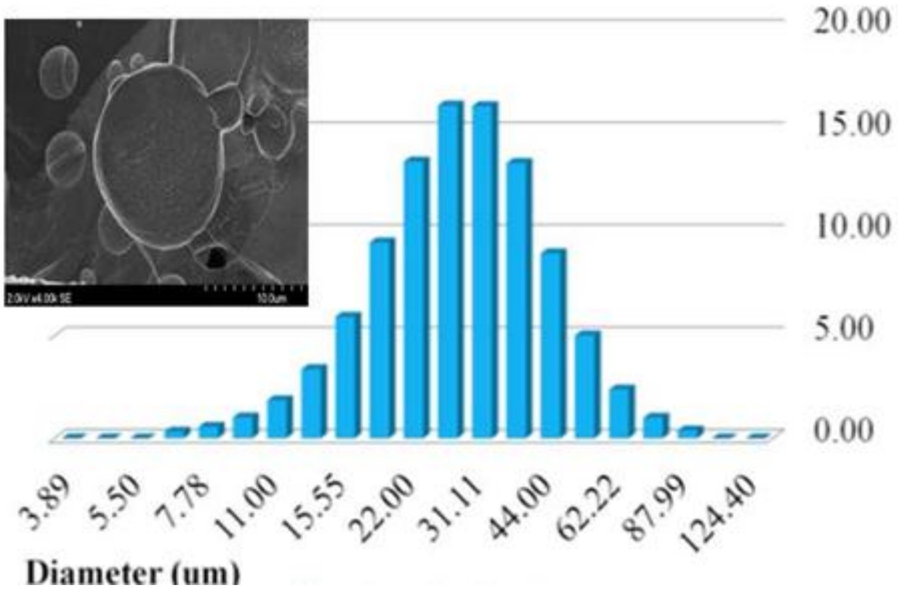

Figure S3 Size distribution of *P. agglomerans*-containing microparticles.

Size distribution of *P. agglomerans*-alginate microcapsules generated from a modified aerosolization/coacervation process. (Inset) Electron micrograph of calcium-alginate encapsulated EGFP *P. agglomerans* E325, size = roughly 10um (center) with 3-4µm microcapsules encapsulating single bacteria.
