# Supplementary figures and images for: A delivery system for field application of paratransgenic control
Source: BMC Biotechnol. 2015 Jun 23;15:59. doi: 10.1186/s12896-015-0175-3 (PMC4477610; doi:10.1186/s12896-015-0175-3)

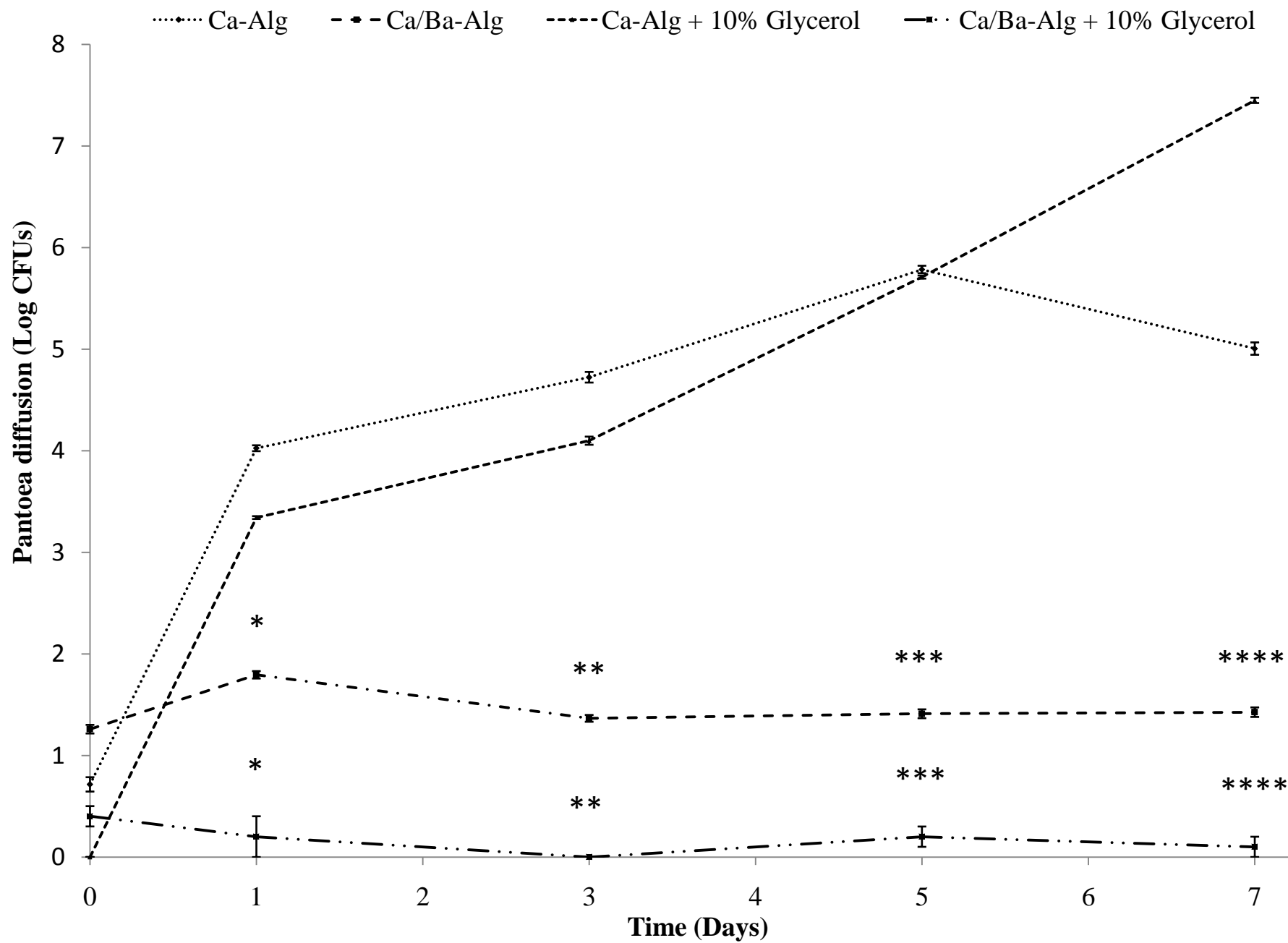

Supplement: Additional file 2: Figure S4. — Diffusion of microencapsulated P. agglomerans from Ca2+ and Ca2+/Ba2+ cross-linked microparticles. Use of barium as a cross-linker greatly reduced the diffusion of bacteria from the microparticles.*, **, *** and **** p < 0.001 by one-way ANOVA with Tukey Simultaneous Tests for means with unequal variance compared to Ca-Alg and Ca-Alg + 10 % Glycerol microparticles on days 1, 3, 5 and 7, respectively. [file 12896_2015_175_MOESM2_ESM.pdf]
