# Supplementary material for: A delivery system for field application of paratransgenic control
Source: BMC Biotechnol. 2015 Jun 23;15:59. doi: 10.1186/s12896-015-0175-3 (PMC4477610; doi:10.1186/s12896-015-0175-3)
Supplement: Additional file 3: Figure S2. — Loss of plasmid pT3078-5 by P. agglomerans over time. [file 12896_2015_175_MOESM3_ESM.pdf]

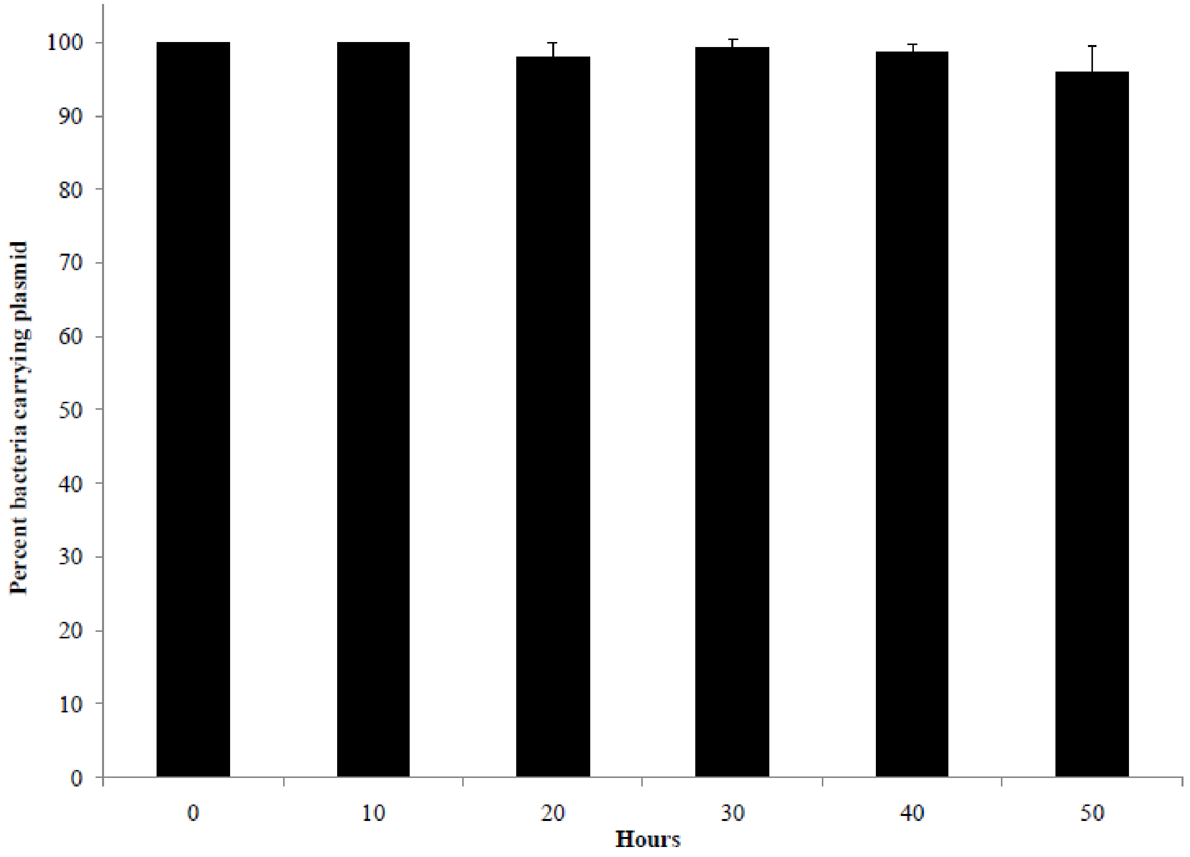

Figure S2 Transformed *P. agglomerans* revert to its wild type form over time.

Percent *P. agglomerans* carrying EGFP-containing plasmid pT3078 in a culture kept at mid log phase for 50 hours (~75 generation). *P. agglomerans* lost pT3078 plasmid at a rate of 0.0533 plasmids per generation.
