# Supplementary material for: A delivery system for field application of paratransgenic control
Source: BMC Biotechnol. 2015 Jun 23;15:59. doi: 10.1186/s12896-015-0175-3 (PMC4477610; doi:10.1186/s12896-015-0175-3)
Supplement: Additional file 4: Figure S1. — Comparison of growth of wild type P. agglomerans and EGFP-expressing P. agglomerans. [file 12896_2015_175_MOESM4_ESM.pdf]

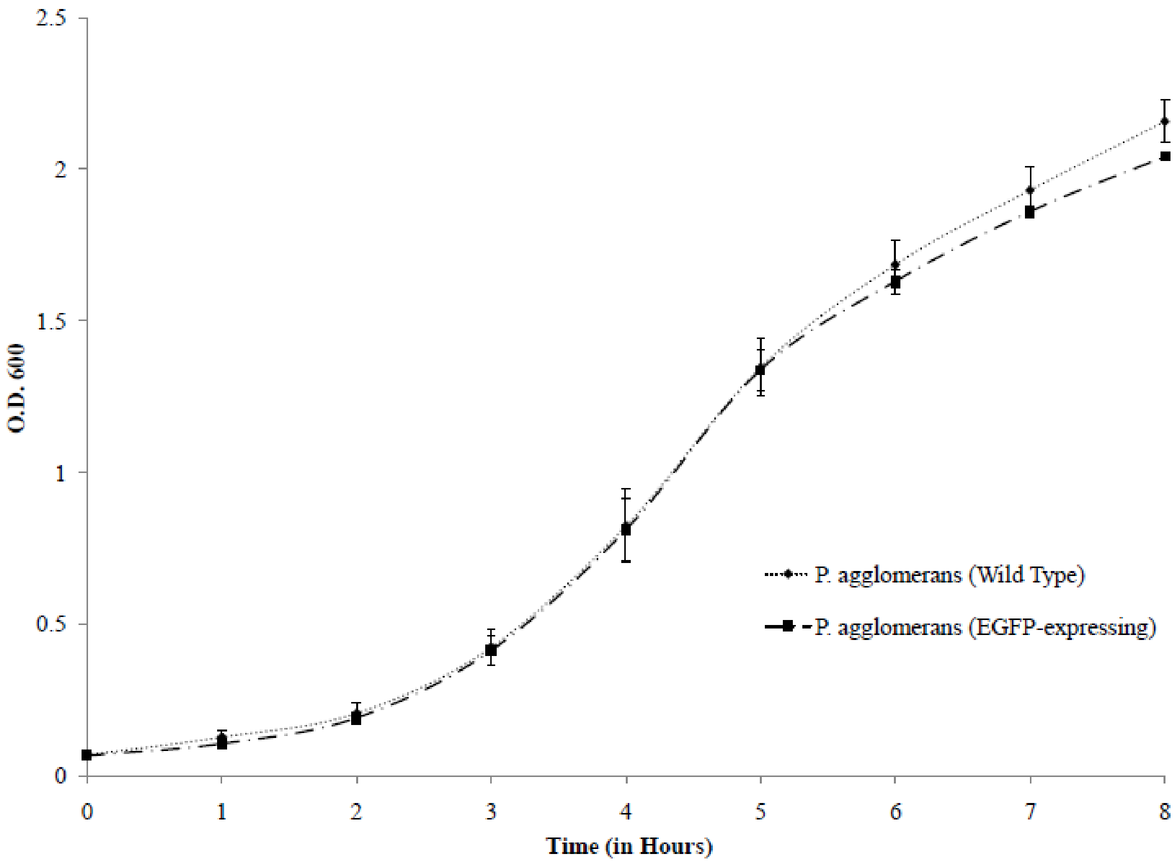

Figure S1 Plasmid pT3078-5 did not affect growth of *P. agglomerans*.

Both the wild type and transformed *P. agglomerans* grew at a similar rate, which shows that the plasmid pT3078-5 did not negatively affect growth of the transformed bacteria.
